# Circulating neurohormone imbalances in canine sudden acquired retinal degeneration syndrome and canine pituitary‐dependent hypercortisolism

**DOI:** 10.1111/jvim.15646

**Published:** 2019-10-29

**Authors:** Annie Oh, Melanie L. Foster, Katharine F. Lunn, Freya M. Mowat

**Affiliations:** ^1^ Department of Clinical Sciences North Carolina State University College of Veterinary Medicine Raleigh North Carolina

**Keywords:** dog, dopamine, melatonin, PDH, retina, SARDS, serotonin

## Abstract

**Background:**

Sudden acquired retinal degeneration syndrome (SARDS) has clinical similarity to pituitary‐dependent hypercortisolism (PDH) in dogs. Some studies have identified a greater frequency of SARDS in seasons with reduced daylight hours. Neurohormone imbalances contribute to retinal lesions in other species, warranting further study in dogs with SARDS.

**Hypothesis:**

Dysregulation of circulating melatonin concentration is present in dogs with SARDS but not in dogs with PDH.

**Animals:**

Fifteen client‐owned dogs with spontaneous SARDS (median time of vision loss 18 days), 14 normal dogs, and 13 dogs with confirmed PDH.

**Procedures:**

Prospective case‐control study. ELISA on samples (obtained in the morning) for measurement of plasma melatonin and dopamine, serum serotonin, urine 6‐sulfatoxymelatonin (MT6s), and creatinine. Statistical analysis was performed using 1‐way ANOVA, Spearman correlation and receiver operator characteristic area under the curve analysis.

**Results:**

There were no significant differences in circulating melatonin, serotonin or dopamine concentrations between the 3 groups, although the study was underpowered for detection of significant differences in serum serotonin. Urine MT6s:creatinine ratio was significantly higher in dogs with PDH (4.08 ± 2.15 urine [MT6s] ng/mL per mg of urine creatinine) compared with dogs with SARDS (2.37 ± .51, *P* < .01), but not compared with normal dogs.

**Conclusions and Clinical Relevance:**

We have identified neurohormone differences between dogs with SARDS and PDH.

**Abbreviations**
AUC: area under the curve
CPT: cell preparation tube
ERG: electroretinography
MT6s: 6‐sulfatoxymelatonin
OCT: spectral‐domain optical coherence tomography
PDH: pituitary‐dependent hypercortisolism
ROC: receiver operator characteristic
SARDS: sudden acquired retinal degeneration syndrome
USG: urine specific gravity

## 1 INTRODUCTION

Sudden acquired retinal degeneration syndrome (SARDS) is a leading cause of acute incurable vision loss in dogs. The pathogenesis of SARDS is poorly understood and there are currently no proven safe and effective treatments.^1^, ^2^, ^3^, ^4^, ^5^ There are existing hypotheses for the etiology of SARDS including autoimmunity^6^, ^7^, ^8^, ^9^, ^10^ and intoxication,^11^ however, a recent clinical trial failed to show that oral immunosuppressive medication was effective in restoring vision.^12^ Thus, exploration of novel hypotheses is warranted to facilitate more effective approaches to treatment. Advancement of our understanding of the disease pathogenesis might also allow identification of useful diagnostic biomarkers for SARDS, allowing earlier therapeutic intervention.

The central hypothesis of this study was that an imbalance of neurohormones is associated with SARDS. There are primary observations underlying this hypothesis, as there is an increased incidence of SARDS cases in seasons with reduced daylight hours.^13^, ^14^ Melatonin influences, and fluctuates with, circadian rhythm and seasonal biology and therefore might explain the seasonal incidence of SARDS.^15^, ^16^, ^17^, ^18^ Furthermore, the retina contains receptors for melatonin.^19^, ^20^, ^21^, ^22^ Although the pineal gland is the primary source of melatonin, it is also produced by other organs. In the retina, melatonin is almost exclusively produced by photoreceptor cells,^21^, ^23^ with melatonin dysregulation proposed to contribute to photoreceptor apoptosis observed in SARDS.^24^ Urine 6‐sulfatoxymelatonin (MT6s) concentrations represent accumulated amounts of systemic melatonin over several hours and are therefore less affected by fluctuations in circulating concentrations. Additionally, urine MT6s accounts for more than 70% of the melatonin secreted, and its concentration in urine is 2‐3 orders of magnitude higher than that of urine melatonin.^25^, ^26^ Related monoamines, serotonin and dopamine, might also be associated with fluctuations in melatonin as serotonin is a precursor to melatonin,^27^, ^28^, ^29^ and dopamine is involved in a cellular feedback loop with melatonin in the retina.^30^, ^31^, ^32^

The purpose of this study was to provide baseline measurements of selected systemic neurohormone concentrations to determine whether this hypothesis merits future mechanistic investigation and whether neurohormone assays might aid in the early diagnosis of SARDS. We measured plasma melatonin, serum serotonin, plasma dopamine, and urine MT6s:creatinine ratio in dogs with SARDS, normal dogs, and dogs with pituitary‐dependent hypercortisolism (PDH). We included dogs with PDH as this condition shares some clinical signs in common with SARDS, and comparison with this group could potentially provide insight into the pathogenesis of some of the systemic clinical signs, such as polyphagia and polyuria‐polydipsia, in dogs with SARDS.

## 2 MATERIALS AND METHODS

### 2.1 Study design

A prospective case‐control study was designed to compare concentrations of morning‐sampled plasma melatonin, serum serotonin, plasma dopamine, urine MT6s, and urine creatinine between client‐owned dogs with confirmed SARDS, normal dogs, and dogs with PDH. A prospective power analysis was performed on available data to calculate sample size to detect a specific change in plasma melatonin concentration. Normal dogs have a serum melatonin mean level of 2.39 ± 0.18 pg/mL (SEM) using data obtained from a group of 19 dogs living in New York State.^33^ Therefore, to detect a proportional difference in melatonin levels comparable to those seen in postmenopausal women with obesity,^34^ at a power of 0.8 and alpha of 0.05, we would need to observe a level of 1.74 pg/mL in our SARDS population compared with normal dogs resulting in a minimum sample size of 12 animals per group. This study was conducted with full institutional approval (Institutional Animal Care and Use Committee approval) and client written informed consent. Full details of recruitment and inclusion/exclusion criteria for the dogs used in this study are described in a previously published study.^35^

### 2.2 Clinical evaluation

All dogs underwent full physical and ophthalmic examination, electroretinography (ERG), spectral‐domain optical coherence tomography (OCT), fasted CBC, serum biochemistry, urinalysis, and ACTH (cosyntropin) stimulation testing as previously described.^12^, ^35^

### 2.3 Sampling and analysis

Voided first morning urine was collected by the owner (approximately 5‐8 am) and samples were stored at −80°C until analysis of MT6s. Fasted blood samples were drawn (between 8 and 9 am) by venipuncture from the jugular vein into 8 mL glass Becton Dickinson Vacutainer Mononuclear Cell Preparation Tubes (CPT, Becton Dickinson Vacutainer, Fischer Scientific, Pittsburg, Pennsylvania) and 4 mL plastic serum blood collection tubes. The CPT tubes were centrifuged at 1500*g* for 20 minutes in a refrigerated centrifuge maintained at 20**°**C according to manufacturer's recommendation. The serum blood collection tube samples were allowed to clot at room temperature for 30 minutes, with shielding from light, and were then centrifuged at 1500*g* for 10 minutes in a refrigerated centrifuge maintained at 20°C. Serum was collected, and stored at −80°C. Samples were not subject to freeze‐thaw cycles before analysis.

Melatonin was extracted from plasma using manufacturer‐supplied extraction columns (Sep‐pak C18 Vac cartridges with a hydrophobic, reverse‐phase, silica‐based bonded phase: KEME761, IBL International, Hamburg, Germany) and an evaporator centrifuge, according to manufacturer's instructions. Validated and commercially available human ELISA kits were used for all neurohormone assays (Table 1). Although these kits were not previously validated for use with canine serum or plasma, human and canine melatonin are predicted to be identical,^36^ and it was anticipated that assays developed for human samples would also be suitable for canine samples, as previously described for a melatonin radioimmunoassay.^33^ In accordance with recent recommendations for reporting of neurohormone assays in publications,^37^ we calculated inter and intra‐assay coefficients of variation for canine samples, and present these data in comparison with manufacturer's published data in Table 1. In addition, any values obtained that were less than the lowest standard were reported as that value and used in statistical analysis.

**Table 1:** Manufacturer supplied and study measured intra‐ and inter‐assay percentage CV. Numbers in parentheses represent the number of samples used for calculation. NA, not applicable (not measured)

Assay	Units of measurement	Manufacturer and catalogue number	Manufacturer datasheet human intra‐assay CV%	Measured canine intra‐assay CV%	Manufacturer datasheet human inter‐assay CV%	Measured canine inter‐assay CV%
Plasma melatonin	pg/mL	IBL international, Hamburg, Germany; RE54021	3‐11.4	22.4 (41)	6.4‐19.3	24.1 (8)
Urine MT6s	ng/mL	ALPCO Diagnostics, Salem, New Hampshire; 79‐STMHU‐E01	5.2‐12.2	8.3 (42)	5.1‐14.9	10.8 (15)
Serum serotonin	ng/mL	ALPCO Diagnostics; 17‐SERHU‐E01‐FST	9.7‐12.6	13.1 (40)	10.4‐12.4	12.7 (24)
Plasma dopamine	pg/mL	ALPCO Diagnostics; 17‐DOPHU‐E01.1	24.4‐29.8	24.0 (42)	14.2‐28.2	NA

Urine creatinine (mg/mL) was quantified using a kinetic colorimetric assay (Jaffe method; Roche Cobas c501 Chemistry analyzer, Indianapolis, Indiana) validated for use in canine clinical samples.

### 2.4 Statistical analysis

One‐way ANOVA with Bonferroni posttest (to correct for type I error) was used to compare dogs with SARDS, normal dogs, and dogs with PDH for the following data sets: daylight duration at time of sampling, post‐ACTH serum cortisol concentration, plasma melatonin concentration, serum serotonin concentration, plasma dopamine concentration, urine specific gravity (USG), creatinine, and MT6s:creatinine ratio. Because of the non‐normal distribution of data (assessed using the D'Agostino and Pearson omnibus normality test), Spearman correlation analysis was completed to model the relationship between serum/plasma neurohormones and post‐ACTH cortisol. Analysis was performed with computerized statistical software (GraphPad Prism version 5.0 for Mac, GraphPad Software, La Jolla, California). Data are presented as mean ± SD and significance was set at *P* < .05.

Receiver operator characteristic (ROC) analysis determines the sensitivity and specificity at different cutoff values for the evaluation of medical diagnostic tests. One index available from an ROC analysis, the area under the curve (AUC) measures the ability of a diagnostic test to discriminate between the “diseased” and “non‐diseased.”^38^, ^39^, ^40^, ^41^ The AUCs were used to analyze the diagnostic ability of plasma melatonin, serum serotonin, plasma dopamine, and urine MT6s:creatinine ratio between groups: SARDS versus normal dogs, SARDS versus PDH, and PDH versus normal dogs. The AUC is classified as follows: .9 to 1.0 = excellent, .80 to .89 = good, .70 to .79 = fair, .60 to .69 = poor, and .50 to .59 = worthless.^42^, ^43^ The best cutoff values were established as the ones for which sensitivity and specificity were maximal (Youden‐Index). The cutoff maximizing the Youden index minimizes the total number of misclassified patients (false positive and false negatives).^41^, ^44^ ROC statistical analyses were performed using MedCalc version 14.12.0 (MedCalc Software bvba, Mariakerke, Belgium).The significance was set at *P* < .05.

## 3 RESULTS

### 3.1 Clinical demographics and examination findings

Clinical demographics, examination, and laboratory findings for the 3 groups were previously described in detail.^35^ All enrolled dogs were included in the analysis. Briefly, 15 dogs with SARDS (8.04 ± 1.7 years) were enrolled and included in data analysis. The median number of days of vision loss at the time of examination was 18 (interquartile range 9 days). No retinal function was identified in any dog in the SARDS group at any electroretinographic (ERG) light intensity (dark‐ and light‐adapted conditions), confirming the diagnosis of SARDS. The control group consisted of 14 normal dogs (8.91 ± 2.68 years; normal retinal function on ERG). The PDH group consisted of 13 dogs (12.0 ± 3.5 years; normal retinal function on ERG). Diagnosis of PDH was based on ACTH stimulation test results, abdominal ultrasound examination that demonstrated bilaterally symmetrically enlarged adrenal glands, endogenous ACTH concentrations and/or results of low‐dose dexamethasone suppression testing. There was a significant difference in post‐ACTH serum cortisol concentrations between groups (1‐way ANOVA *P* < .0001). Bonferroni posttest showed that dogs with PDH had a significantly higher concentration of serum cortisol post‐ACTH (30.9 ± 8.9 μg/dL) compared with both normal dogs (15.2 ± 2.6 μg/dL *P* < .0001) and dogs with SARDS (17.4 ± 4.1 μg/dL, *P* < .0001). There were no differences in post‐ACTH serum cortisol concentrations between normal dogs and dogs with SARDS.

The season of recruitment was not controlled for during the enrollment for the study. Dogs with SARDS were recruited over a 1 year period (4/15 spring March‐May, 2/15 summer June‐August, 6/15 fall September‐November, 3/15 winter; December‐February). Dogs with PDH were recruited over a 1 year and 2 month period (7/13 spring, 3/13 summer, 2/13 fall, and 1/13 winter). Normal dogs were all recruited in either spring (9/14) or fall (5/14). There was a significant variation in daylight duration (source aa.usno.navy.mil) on the date of enrollment between groups using 1‐way ANOVA (*P* = .04). Post hoc Bonferroni test identified that dogs with PDH were experiencing a significantly longer daylight duration (779 ± 80 minutes) compared with dogs with SARDS (696 ± 91 minutes, *P* < .05). There was no significant difference between normal dogs (741 ± 85 minutes) and dogs with either SARDS or PDH.

### 3.2 Plasma melatonin and urine 6‐sulfatoxymelatonin:creatinine ratio

**Figure 1:**
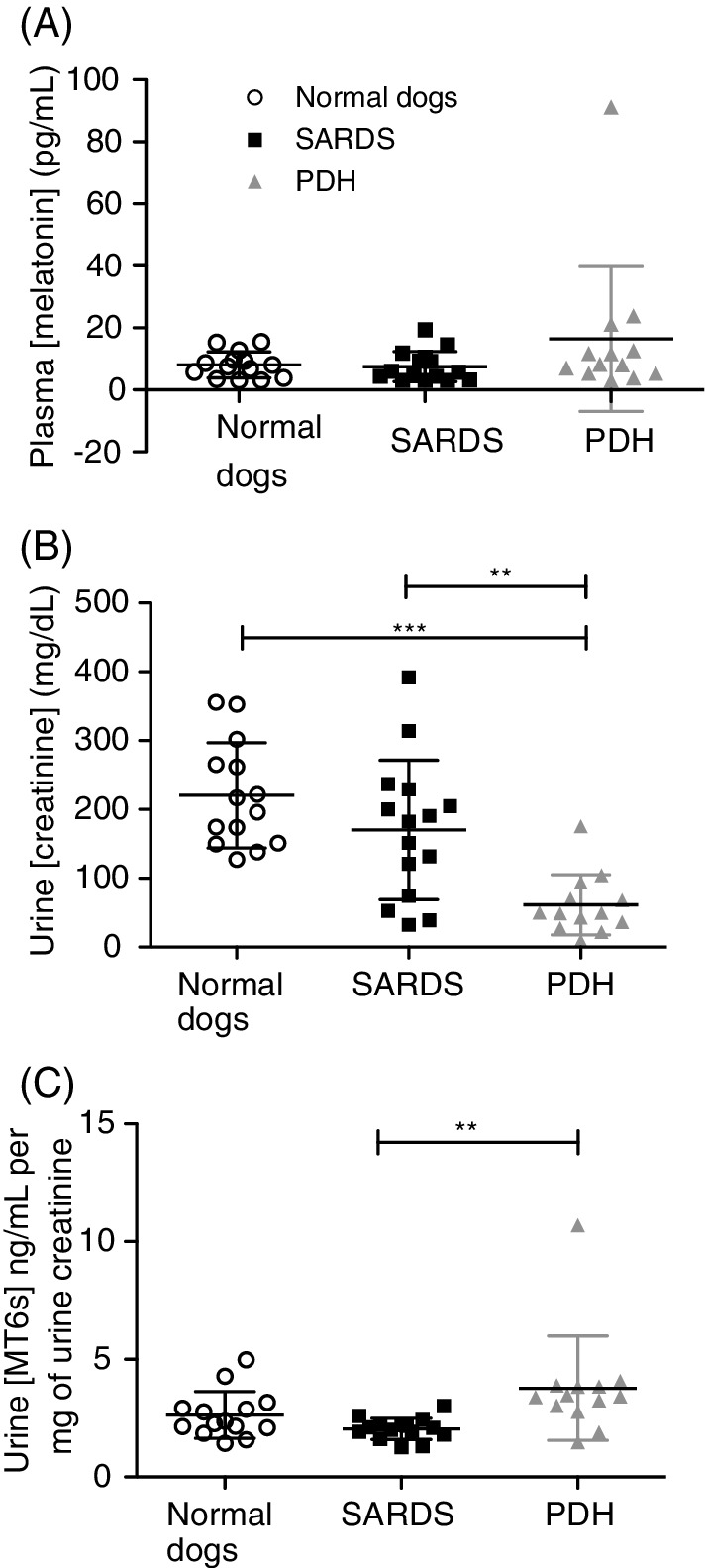
Vertical scatter plots showing plasma melatonin, urine creatinine, and urine 6‐sulfatoxymelatonin concentrations. A, There were no significant differences in plasma melatonin concentrations between groups. B, Dogs with PDH had significantly lower urine creatinine concentrations than both of the other groups, reflecting a reduction in urine specific gravity. C, Dogs with PDH had significantly higher urine MT6s:creatinine ratios compared to dogs with SARDS. Each column in graphs represents mean value (horizontal line in the middle) ± SD. Lines connecting columns represent significantly different comparisons, 1‐way ANOVA with Bonferroni posttest **P < .01, ***P < .001. MT6s, 6‐sulfatoxymelatonin; PDH, pituitary‐dependent hypercortisolism; SARDS, sudden acquired retinal degeneration syndrome

When 1‐way ANOVA was performed on data from all dogs, there were no significant differences in plasma melatonin concentration between dogs with SARDS (7.49 ± 4.88 pg/mL), normal dogs (8.04 ± 4.20 pg/mL), and dogs with PDH (16.4 ± 23.32 pg/mL, overall *P* = .17, Figure 1A).

Urine creatinine was significantly different between groups using 1‐way ANOVA (*P* < .0001). Bonferroni posttest identified dogs with PDH had significantly lower urine creatinine (61.4 ± 43.7 mg/dL) concentrations compared with both dogs with SARDS (170.2 ± 101.3 mg/dL, *P* < .01) and normal dogs (220.5 ± 76.6 mg/dL, *P* < .001, Figure 1B), reflecting less concentrated urine in this group. Urine specific gravity was also significantly different between groups using 1‐way ANOVA (*P* < .0001). Bonferroni posttest identified that dogs with PDH had significantly lower USG (1.012 ± .007) compared to dogs with SARDS (1.031 ± .0132; *P* < .001), and USG was also significantly lower in dogs with PDH compared to normal dogs (1.039 ± .008; *P* < .001). There was no significant difference in USG between dogs with SARDS and normal dogs.

Urine MT6s:creatinine ratio was significantly different between the 3 groups using 1‐way ANOVA (*P* = .009). Bonferroni posttest identified that urine MT6s:creatinine ratio was significantly higher in dogs with PDH (4.08 ± 2.15 urine [MT6s] ng/mL per mg of urine creatinine) compared to dogs with SARDS (2.37 ± .51 urine [MT6s] ng/mL per mg of urine creatinine, *P* < .01, Figure 1C). There was no difference in the MT6s:creatinine ratio between dogs with either SARDS or PDH and normal dogs (3.11 ± 1.14 urine [MT6s] ng/mL per mg of urine creatinine, *P* > .05, Figure 1C). Based on AUC values, the urine MT6s:creatinine ratio had “good” diagnostic ability to discriminate between dogs with SARDS and dogs with PDH (AUC: .856, at >3.047 ng/mL per mg of urine creatinine sensitivity: 84.6%, specificity: 93.3%), but only had “fair” diagnostic ability to differentiate dogs with SARDS from normal dogs (AUC: .714, at <2.363 ng/mL per mg of urine creatinine sensitivity: 60.0%, specificity: 78.6%), and “poor” diagnostic ability to discriminate between normal dogs and dogs with PDH (AUC: .681, at >3.288 ng/mL per mg of urine creatinine sensitivity: 76.9%, specificity: 71.4%; Table 2).

**Table 2:** Area under the receiver operating characteristic curves (AUCs) and 95% confidence intervals (CIs) for each neurohormone parameter to discriminate between dogs with sudden acquired retinal degeneration syndrome (SARDS), normal dogs, and dogs with pituitary‐dependent hypercortisolism (PDH)

Variables	Units	AUC (95% CI)
Comparison 1: normal dogs versus SARDS		
Melatonin	pg/mL	.556 (.342‐.772)
6‐sulfatoxymelatonin:creatinine ratio	ng/mL/mg	.714 (.520‐.909)
Serotonin	ng/mL	.724 (.530‐.918)
Dopamine	pg/mL	.538 (.325‐.751)
Comparison 2: SARDS versus PDH		
Melatonin	pg/mL	.656 (.450‐.862)
6‐sulfatoxymelatonin:creatinine ratio	ng/mL/mg	.856 (.682‐1.030)
Serotonin	ng/mL	.697 (.494‐.901)
Dopamine	pg/mL	.513 (.288‐.739)
Comparison 3: normal dogs versus PDH		
Melatonin	pg/mL	.599 (.380‐.818)
6‐sulfatoxymelatonin:creatinine ratio	ng/mL/mg	.681 (.463‐.900)
Serotonin	ng/mL	.522 (.296‐.748)
Dopamine	pg/mL	.529 (.309‐.748)

**Figure 2:**
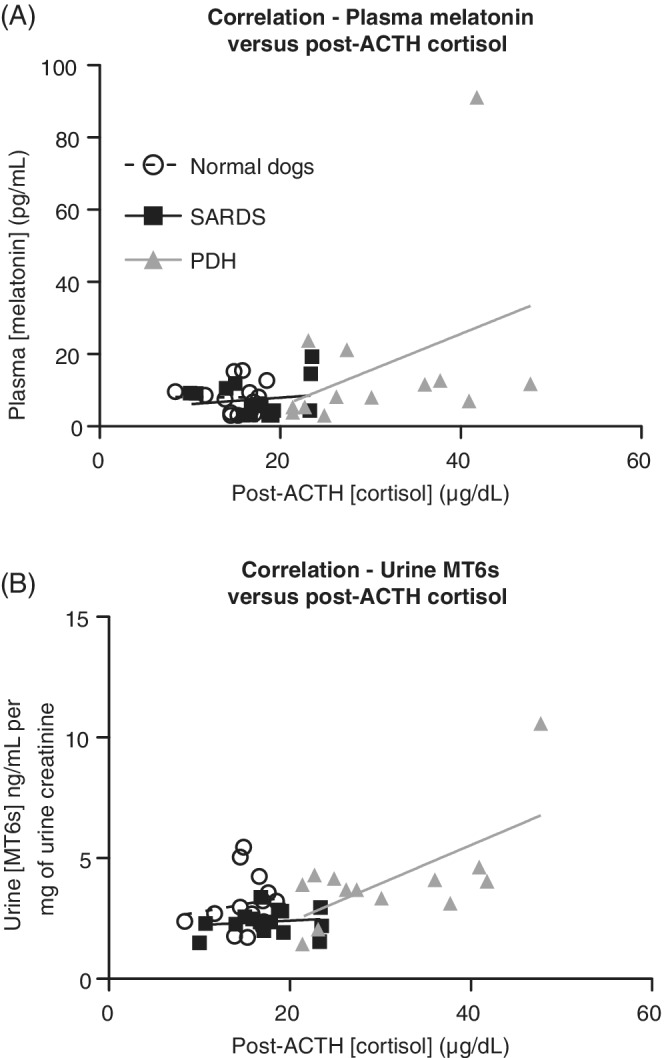
X‐Y scatter plot showing correlation between melatonin assays and post‐ACTH cortisol concentrations. A, Linear regression analysis showed significant correlation between plasma melatonin and post‐ACTH serum cortisol concentration. B, Linear regression analysis showed significant correlation between urine MT6s:creatinine ratio and post‐ACTH serum cortisol concentration. MT6s, 6‐sulfatoxymelatonin; PDH, pituitary‐dependent hypercortisolism; SARDS, sudden acquired retinal degeneration syndrome

Spearman correlation analysis determined that when including all groups together, post‐ACTH serum cortisol and plasma melatonin were not significantly correlated (Spearman *r* = .19, *P* = .23). However, when groups were considered separately, post‐ACTH serum cortisol and plasma melatonin were significantly positively correlated in dogs with PDH (*r* = .56, *P* = .05) but not in normal dogs (*r* = .02, *P* = .94) or dogs with SARDS (*R* = −.07, *P* = .80). Spearman correlation analysis determined that when including all groups together, post‐ACTH serum cortisol and urine MT6s:creatinine ratio were positively correlated (Spearman *r* = .39, *P* = .0098; Figure 2B). When groups were considered separately, post‐ACTH serum cortisol and urine MT6s:creatinine ratio were not significantly correlated in dogs with PDH (*r* = .50, *P* = .08), in normal dogs (*r* = .13, *P* = .65) and dogs with SARDS (*r* = .03, *P* = .92).

### 3.3 Serum serotonin

**Figure 3:**
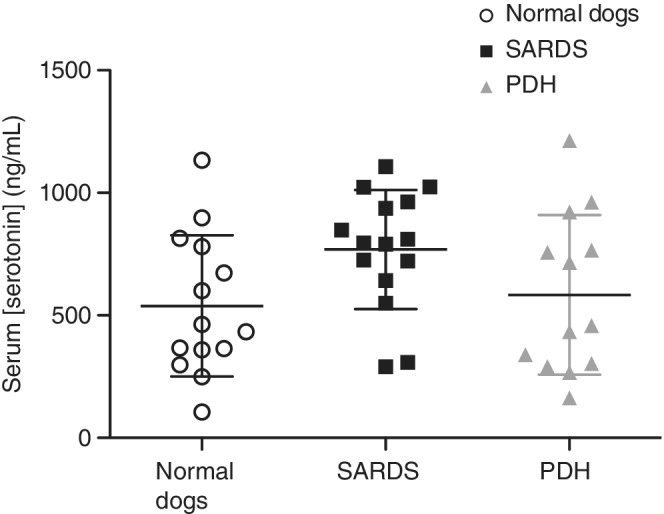
Vertical scatter plots showing serum serotonin concentrations. Each column in graphs represents mean value (horizontal line in the middle) ± SD. PDH, pituitary‐dependent hypercortisolism; SARDS, sudden acquired retinal degeneration syndrome

There were no significant differences in the concentration of serum serotonin between dogs with SARDS (768.4 ± 242.9 ng/mL), normal dogs (525.2 ± 295.3 ng/mL), and dogs with PDH (581.8 ± 322.5 ng/mL, overall *P* = .08, Figure 3A). The original study sample size calculation was made based on canine plasma melatonin, therefore we performed a post hoc power analysis using our generated serotonin ELISA data from dogs with SARDS and controls, to form the basis for future studies. Based on the observed variation and effect size, an alpha of .05 and 80% power, a sample size of 16 animals per group would be needed to detect a significant difference in serum serotonin, indicating that the serotonin assay was underpowered to detect a significant difference, Serum serotonin values did not correlate significantly with post‐ACTH cortisol in either dogs with SARDS or dogs with PDH (data not shown).

### 3.4 Plasma dopamine

**Figure 4:**
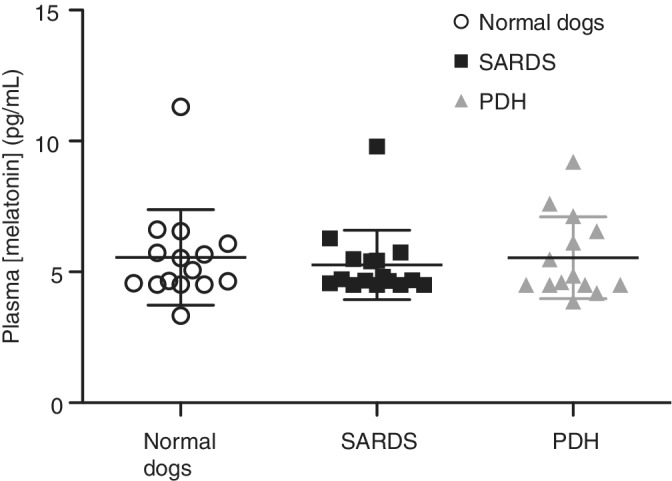
Vertical scatter plots showing plasma dopamine concentrations. There were no significant differences in the concentrations of dopamine in plasma between normal dogs, dogs with SARDS, and dogs with PDH. Each column in graphs represents mean value (horizontal line in the middle) ± SD. PDH, pituitary‐dependent hypercortisolism; SARDS, sudden acquired retinal degeneration syndrome

Using 1‐way ANOVA, there were no significant differences in the concentration of dopamine in plasma between dogs with SARDS (5.27 ± 1.32 pg/mL), normal dogs (5.54 ± 1.82 pg/mL), and dogs with PDH (5.54 ± 1.56 pg/mL, *P* = .86, Figure 4). Plasma dopamine values did not correlate significantly with post‐ACTH cortisol in either dogs with SARDS or dogs with PDH (data not shown).

## 4 DISCUSSION

This study evaluated differences in melatonin, serotonin, and dopamine between dogs with SARDS, normal dogs, and dogs with PDH. Urine MT6s:creatinine ratio was higher in dogs with PDH compared to dogs with SARDS. Serum serotonin concentrations were higher in dogs with SARDS, although differences were not significant between the groups. No differences were noted in plasma dopamine concentrations among the 3 groups. The differences in neurohormone concentrations between groups found in this study suggest that future research is warranted in this area, and might also propose new directions for therapeutic strategies.

Our observation that plasma melatonin correlates with post‐ACTH cortisol in dogs with PDH raises some interesting questions. The initial motivation was to study SARDS and utilize PDH as a comparison group because these 2 diseases have shared clinical features.^1^, ^2^, ^3^, ^35^, ^45^, ^46^ It is likely that our results were affected by the season of recruitment, as daylight duration was significantly longer in dogs with PDH compared to dogs with SARDS. However, considering that melatonin in dogs peaks in winter (a season with shorter daylight duration),^33^ our results most likely represent a relative underestimate of the melatonin levels in dogs with PDH. Melatonin has been proposed as a therapeutic agent for atypical hypercortisolism in dogs. A study performed in dispersed cells and explants from primate adrenal glands showed that melatonin inhibited corticotropin (ACTH)‐stimulated cortisol production, possibly through a melatonin receptor, MT1.^47^ Others showed in cell cultures of human adrenocortical carcinoma cells, that combined lignan phytoestrogen and melatonin treatment decreased the secretion of cortisol.^48^ However, in our study, morning plasma melatonin trended toward increased values, and morning urine MT6s concentrations were significantly increased in dogs with PDH and not in dogs with SARDS when compared to normal dogs. This is further supported by correlation data showing that circulating melatonin is significantly correlated with post‐ACTH serum cortisol, indicating that dogs with greater circulating melatonin have a more robust response to exogenous ACTH. This suggests that dogs with increased steroidogenesis do not lack melatonin. This finding therefore questions the value of melatonin supplementation in dogs with PDH. Further research is required to determine cortisol‐melatonin interactions and the mechanisms regulating melatonin in dogs with hypercortisolism. We however acknowledge that a limitation of this study was that the neurohormone measurements were made only at a single morning time point. Melatonin fluctuates in dogs in response to circadian rhythm, although peak amounts are detected in the early morning.^33^ In addition, we were not able to generate sufficient statistical power to establish if melatonin has seasonal variation in either dogs with PDH or SARDS and further work is needed to examine the effect of day and season on circulating melatonin in PDH and SARDS. Because multiple comparisons were performed in this study, there was also the potential for inflated type I error.

Our study was underpowered for detection of a significant difference in circulating serotonin concentrations between normal dogs and dogs with SARDS. Future studies with a larger sample size are needed to confirm if dogs with SARDS have more circulating serotonin than controls. Potential explanations for increased circulating serotonin are either gastrointestinal or hematological. Approximately 90%‐95% of serotonin in the body is stored in intestinal enterochromaffin cells.^49^, ^50^, ^51^, ^52^ Carcinoids arising from enterochromaffin cells often secrete serotonin and the serotonin precursor 5‐hydroxytryptophan.^53^, ^54^ There is no evidence for systemic neoplasia contributing to SARDS pathogenesis,^7^ therefore enteric carcinoid tumor formation as a cause of elevated circulating serotonin is unlikely. Approximately 5%‐8% of body serotonin is stored in platelets, and circulating blood serotonin is almost exclusively stored and transported in platelet dense granules.^55^ In response to damaged endothelium and ischemia, platelets aggregate and release serotonin into the bloodstream,^56^ contributing to thrombus formation and vasoconstriction.^57^ We hypothesize that platelet aggregation and activation leading to the release of serotonin could contribute to the pathogenesis of SARDS. It is possible that this exacerbates retinal disease in SARDS by inducing vasoconstriction in retinal vasculature. In experimental primate, porcine, and murine models, exogenous serotonin induced vasoconstriction at the level of the caudal ocular segment.^58^, ^59^, ^60^ Furthermore, in primates with atherosclerotic lesions, infusion of serotonin resulted in vasospasm of the central retinal artery and/or caudal ciliary artery^61^ and profoundly decreased retinal ERG responses.^62^ The potential for serotonin, platelet aggregation and ischemia as key players in the pathogenesis of SARDS warrants further investigation.

## CONFLICT OF INTEREST DECLARATION

Authors declare no conflict of interest.

## OFF‐LABEL ANTIMICROBIAL DECLARATION

Authors declare no off‐label use of antimicrobials.

## INSTITUTIONAL ANIMAL CARE AND USE COMMITTEE (IACUC) OR OTHER APPROVAL DECLARATION

Approval by the North Carolina State University IACUC, protocol number 15‐132‐O.

## HUMAN ETHICS APPROVAL DECLARATION

Authors declare human ethics approval was not needed for this study.

## References

[1] Carter RT , Oliver JW , Stepien RL , Bentley E . Elevations in sex hormones in dogs with sudden acquired retinal degeneration syndrome (SARDS). J Am Anim Hosp Assoc. 2009;45:207‐214. 19723843 10.5326/0450207

[2] Stuckey JA , Pearce JW , Giuliano EA , et al. Long‐term outcome of sudden acquired retinal degeneration syndrome in dogs. J Am Vet Med Assoc. 2013;243:1425‐1431. 24171371 10.2460/javma.243.10.1426

[3] Komaromy AM , Abrams KL , Heckenlively JR , et al. Sudden acquired retinal degeneration syndrome (SARDS)—a review and proposed strategies toward a better understanding of pathogenesis, early diagnosis, and therapy. Vet Ophthalmol. 2016;19:319‐331. 26096588 10.1111/vop.12291

[4] Heller AR , van der Woerdt A , Gaarder JE , et al. Sudden acquired retinal degeneration in dogs: breed distribution of 495 canines. Vet Ophthalmol. 2017;20:103‐106. 26938661 10.1111/vop.12370

[5] Montgomery KW , van der Woerdt A , Cottrill NB . Acute blindness in dogs: sudden acquired retinal degeneration syndrome versus neurological disease (140 cases, 2000‐2006). Vet Ophthalmol. 2008;11:314‐320. 19046291 10.1111/j.1463-5224.2008.00652.x

[6] Braus BK , Hauck SM , Amann B , et al. Neuron‐specific enolase antibodies in patients with sudden acquired retinal degeneration syndrome. Vet Immunol Immunopathol. 2008;124:177‐183. 18405980 10.1016/j.vetimm.2008.02.020

[7] Gilmour MA , Cardenas MR , Blaik MA , Bahr RJ , McGinnis JF . Evaluation of a comparative pathogenesis between cancer‐associated retinopathy in humans and sudden acquired retinal degeneration syndrome in dogs via diagnostic imaging and western blot analysis. Am J Vet Res. 2006;67:877‐881. 16649924 10.2460/ajvr.67.5.877

[8] Keller RL , Kania SA , Hendrix DV , Ward DA , Abrams K . Evaluation of canine serum for the presence of antiretinal autoantibodies in sudden acquired retinal degeneration syndrome. Vet Ophthalmol. 2006;9:195‐200. 16634935 10.1111/j.1463-5224.2006.00466.x

[9] Grozdanic SD , Lazic T , Kecova H , Mohan K , Kuehn MH . Optical coherence tomography and molecular analysis of sudden acquired retinal degeneration syndrome (SARDS) eyes suggests the immune‐mediated nature of retinal damage. Vet Ophthalmol. 2019;22:305‐327. 30109754 10.1111/vop.12597 PMC6563498

[10] Bellhorn RW , Murphy CJ , Thirkill CE . Anti‐retinal immunoglobulins in canine ocular diseases. Semin Vet Med Surg (Small Anim). 1988;3:28‐32. 3363244

[11] Vainisi SJ , Schmidt GM , West CS , et al. Metabolic toxic retinopathy: preliminary report. Trans Am Coll Vet Ophthalmol. 1983;14:6.

[12] Young WM , Oh A , Williams JG , et al. Clinical therapeutic efficacy of mycophenolate mofetil in the treatment of SARDS in dogs‐a prospective open‐label pilot study. Vet Ophthalmol. 2018;21:565‐576. 29383824 10.1111/vop.12545

[13] Acland GM , Aguirre GD . Sudden acquired retinal degeneration: clinical signs and diagnosis. Trans Am Coll Vet Ophthalmol. 1986;17:6.

[14] Acland GM , Irby NL , Aguirre GD , et al. Sudden acquired retinal degeneration in the dog: clinical and morphologic characterization of the “silent retina” syndrome. Trans Am Coll Vet Ophthalmol. 1984;15:18.

[15] Reiter RJ . The melatonin rhythm: both a clock and a calendar. Experientia. 1993;49:654‐664. 8395408 10.1007/BF01923947

[16] Reiter RJ . Melatonin: the chemical expression of darkness. Mol Cell Endocrinol. 1991;79:C153‐C158. 1936532 10.1016/0303-7207(91)90087-9

[17] Reiter RJ . The pineal and its hormones in the control of reproduction in mammals. Endocr Rev. 1980;1:109‐131. 6263600 10.1210/edrv-1-2-109

[18] Malpaux B , Thiery JC , Chemineau P . Melatonin and the seasonal control of reproduction. Reprod Nutr Dev. 1999;39:355‐366. 10420438 10.1051/rnd:19990308

[19] Tosini G , Baba K , Hwang CK , Iuvone PM . Melatonin: an underappreciated player in retinal physiology and pathophysiology. Exp Eye Res. 2012;103:82‐89. 22960156 10.1016/j.exer.2012.08.009 PMC3462291

[20] Wiechmann AF , Summers JA . Circadian rhythms in the eye: the physiological significance of melatonin receptors in ocular tissues. Prog Retin Eye Res. 2008;27:137‐160. 18316227 10.1016/j.preteyeres.2007.10.001

[21] Tosini G , Menaker M . The clock in the mouse retina: melatonin synthesis and photoreceptor degeneration. Brain Res. 1998;789:221‐228. 9573370 10.1016/s0006-8993(97)01446-7

[22] Tosini G , Owino S , Guillaume JL , Jockers R . Understanding melatonin receptor pharmacology: latest insights from mouse models, and their relevance to human disease. Bioessays. 2014;36:778‐787. 24903552 10.1002/bies.201400017 PMC4151498

[23] Liu C , Fukuhara C , Wessel JH 3rd , et al. Localization of Aa‐nat mRNA in the rat retina by fluorescence in situ hybridization and laser capture microdissection. Cell Tissue Res. 2004;315:197‐201. 14618388 10.1007/s00441-003-0822-1

[24] Miller PE , Galbreath EJ , Kehren JC , Steinberg H , Dubielzig RR . Photoreceptor cell death by apoptosis in dogs with sudden acquired retinal degeneration syndrome. Am J Vet Res. 1998;59:149‐152. 9492927

[25] Graham C , Cook MR , Kavet R , Sastre A , Smith DK . Prediction of nocturnal plasma melatonin from morning urinary measures. J Pineal Res. 1998;24:230‐238. 9572533 10.1111/j.1600-079x.1998.tb00538.x

[26] Paakkonen T , Makinen TM , Leppaluoto J , et al. Urinary melatonin: a noninvasive method to follow human pineal function as studied in three experimental conditions. J Pineal Res. 2006;40:110‐115. 16441547 10.1111/j.1600-079X.2005.00300.x

[27] Ganguly S , Coon SL , Klein DC . Control of melatonin synthesis in the mammalian pineal gland: the critical role of serotonin acetylation. Cell Tissue Res. 2002;309:127‐137. 12111543 10.1007/s00441-002-0579-y

[28] Mohammad‐Zadeh LF , Moses L , Gwaltney‐Brant SM . Serotonin: a review. J Vet Pharmacol Ther. 2008;31:187‐199. 18471139 10.1111/j.1365-2885.2008.00944.x

[29] Kirsz K , Zieba DA . A review on the effect of the photoperiod and melatonin on interactions between ghrelin and serotonin. Gen Comp Endocrinol. 2012;179:248‐253. 22974511 10.1016/j.ygcen.2012.08.025

[30] Zawilska JB , Iuvone PM . Melatonin synthesis in chicken retina: effect of kainic acid‐induced lesions on the diurnal rhythm and D2‐dopamine receptor‐mediated regulation of serotonin N‐acetyltransferase activity. Neurosci Lett. 1992;135:71‐74. 1347416 10.1016/0304-3940(92)90138-w

[31] Nguyen‐Legros J , Chanut E , Versaux‐Botteri C , Simon A , Trouvin JH . Dopamine inhibits melatonin synthesis in photoreceptor cells through a D2‐like receptor subtype in the rat retina: biochemical and histochemical evidence. J Neurochem. 1996;67:2514‐2520. 8931485 10.1046/j.1471-4159.1996.67062514.x

[32] Tosini G , Dirden JC . Dopamine inhibits melatonin release in the mammalian retina: in vitro evidence. Neurosci Lett. 2000;286:119‐122. 10825651 10.1016/s0304-3940(00)01117-4

[33] Dunlap KL , Reynolds AJ , Tosini G , Kerr WW , Duffy LK . Seasonal and diurnal melatonin production in exercising sled dogs. Comp Biochem Physiol A Mol Integr Physiol. 2007;147:863‐867. 17379556 10.1016/j.cbpa.2007.02.015

[34] Walecka‐Kapica E , Chojnacki J , Stepien A , et al. Melatonin and female hormone secretion in postmenopausal overweight women. Int J Mol Sci. 2015;16:1030‐1042. 25569084 10.3390/ijms16011030 PMC4307288

[35] Oh A , Foster ML , Williams JG , et al. Diagnostic utility of clinical and laboratory test parameters for differentiating between sudden acquired retinal degeneration syndrome and pituitary‐dependent hyperadrenocorticism in dogs. Vet Ophthalmol. 2019. 10.1111/vop.12661 30864251

[36] Zhao D , Yu Y , Shen Y , et al. Melatonin synthesis and function: evolutionary history in animals and plants. Front Endocrinol (Lausanne). 2019;10:249. 31057485 10.3389/fendo.2019.00249 PMC6481276

[37] Kennaway DJ . A critical review of melatonin assays: past and present. J Pineal Res. 2019;67:e12572. 30919486 10.1111/jpi.12572

[38] Hanley JA . Receiver operating characteristic (ROC) methodology: the state of the art. Crit Rev Diagn Imaging. 1989;29:307‐335. 2667567

[39] Kumar R , Indrayan A . Receiver operating characteristic (ROC) curve for medical researchers. Indian Pediatr. 2011;48:277‐287. 21532099 10.1007/s13312-011-0055-4

[40] Hanley JA , McNeil BJ . The meaning and use of the area under a receiver operating characteristic (ROC) curve. Radiology. 1982;143:29‐36. 7063747 10.1148/radiology.143.1.7063747

[41] Hajian‐Tilaki K . Receiver operating characteristic (ROC) curve analysis for medical diagnostic test evaluation. Caspian J Intern Med. 2013;4:627‐635. 24009950 PMC3755824

[42] Metz CE . Basic principles of ROC analysis. Semin Nucl Med. 1978;8:283‐298. 112681 10.1016/s0001-2998(78)80014-2

[43] Obuchowski NA , Bullen JA . Receiver operating characteristic (ROC) curves: review of methods with applications in diagnostic medicine. Phys Med Biol. 2018;63:07TR01. 10.1088/1361-6560/aab4b1 29512515

[44] Schweitzer C , Korobelnik JF , Le Goff M , et al. Diagnostic performance of peripapillary retinal nerve Fiber layer thickness for detection of glaucoma in an elderly population: the ALIENOR study. Invest Ophthalmol Vis Sci. 2016;57:5882‐5891. 27802518 10.1167/iovs.16-20104

[45] Van der Woerdt A , Nasisse MP , Davidson MG . Sudden acquired retinal degeneration in the dog: clinical and laboratory findings in 36 cases. Prog Vet Comp Ophthalmol. 1991;1:8.

[46] Mattson A , Roberts SM , Isherwood JME . Clinical‐features suggesting hyperadrenocorticism associated with sudden acquired retinal degeneration syndrome in a dog. J Am Anim Hosp Assoc. 1992;28:199‐202.

[47] Torres‐Farfan C , Richter HG , Rojas‐Garcia P , et al. mt1 melatonin receptor in the primate adrenal gland: inhibition of adrenocorticotropin‐stimulated cortisol production by melatonin. J Clin Endocrinol Metab. 2003;88:450‐458. 12519889 10.1210/jc.2002-021048

[48] Fecteau KA , Eiler H , Oliver JW . Effect of combined lignan phytoestrogen and melatonin treatment on secretion of steroid hormones by adrenal carcinoma cells. Am J Vet Res. 2011;72:675‐680. 21529220 10.2460/ajvr.72.5.675

[49] Berger M , Gray JA , Roth BL . The expanded biology of serotonin. Annu Rev Med. 2009;60:355‐366. 19630576 10.1146/annurev.med.60.042307.110802 PMC5864293

[50] Bertrand PP , Bertrand RL . Serotonin release and uptake in the gastrointestinal tract. Auton Neurosci. 2010;153:47‐57. 19729349 10.1016/j.autneu.2009.08.002

[51] Costedio MM , Hyman N , Mawe GM . Serotonin and its role in colonic function and in gastrointestinal disorders. Dis Colon Rectum. 2007;50:376‐388. 17195902 10.1007/s10350-006-0763-3

[52] Gershon MD . Review article: serotonin receptors and transporters—roles in normal and abnormal gastrointestinal motility. Aliment Pharmacol Ther. 2004;20(Suppl 7):3‐14. 10.1111/j.1365-2036.2004.02180.x 15521849

[53] Kema IP , de Vries EG , Schellings AM , Postmus PE , Muskiet FA . Improved diagnosis of carcinoid tumors by measurement of platelet serotonin. Clin Chem. 1992;38:534‐540. 1373675

[54] Janson ET , Holmberg L , Stridsberg M , et al. Carcinoid tumors: analysis of prognostic factors and survival in 301 patients from a referral center. Ann Oncol. 1997;8:685‐690. 9296223 10.1023/a:1008215730767

[55] Maurer‐Spurej E , Pittendreigh C , Solomons K . The influence of selective serotonin reuptake inhibitors on human platelet serotonin. Thromb Haemost. 2004;91:119‐128. 14691577 10.1160/TH03-05-0330

[56] Jonnakuty C , Gragnoli C . What do we know about serotonin? J Cell Physiol. 2008;217:301‐306. 18651566 10.1002/jcp.21533

[57] Cote F , Fligny C , Fromes Y , et al. Recent advances in understanding serotonin regulation of cardiovascular function. Trends Mol Med. 2004;10:232‐238. 15121050 10.1016/j.molmed.2004.03.007

[58] Hayreh SS . Retinal and optic nerve head ischemic disorders and atherosclerosis: role of serotonin. Prog Retin Eye Res. 1999;18:191‐221. 9932283 10.1016/s1350-9462(98)00016-0

[59] Haefliger IO , Flammer J , Luscher TF . Heterogeneity of endothelium‐dependent regulation in ophthalmic and ciliary arteries. Invest Ophthalmol Vis Sci. 1993;34:1722‐1730. 8473112

[60] Boerrigter RM , Siertsema JV , Kema IP . Serotonin (5‐HT) and the rat's eye. Some pilot studies. Doc Ophthalmol. 1992;82:141‐150. 1305018 10.1007/BF00157004

[61] Hayreh SS , Piegors DJ , Heistad DD . Serotonin‐induced constriction of ocular arteries in atherosclerotic monkeys. Implications for ischemic disorders of the retina and optic nerve head. Arch Ophthalmol. 1997;115:220‐228. 9046257 10.1001/archopht.1997.01100150222012

[62] Williams JK , Baumbach GL , Armstrong ML , Heistad DD . Hypothesis: vasoconstriction contributes to amaurosis fugax. J Cereb Blood Flow Metab. 1989;9:111‐116. 2910892 10.1038/jcbfm.1989.15

